# An open-label, randomized bioavailability study with alternative methods of administration of crushed ticagrelor tablets in healthy volunteers

**DOI:** 10.5414/CP202202

**Published:** 2014-12-15

**Authors:** Renli Teng, Glenn Carlson, Judith Hsia

**Affiliations:** AstraZeneca LP, Wilmington, DE, USA

**Keywords:** ticagrelor, drug administration routes, bioequivalence

## Abstract

Objective: To compare the bioavailability and safety profile of crushed ticagrelor tablets suspended in water and administered orally or via nasogastric tube, with that of whole tablets administered orally. Methods: In this single-center, open-label, randomized, three-treatment crossover study, 36 healthy volunteers were randomized to receive a single 90-mg dose of ticagrelor administered orally as a whole tablet or as crushed tablets suspended in water and given orally or via a nasogastric tube into the stomach, with a minimum 7-day wash-out between treatments. Plasma concentrations of ticagrelor and AR-C124910XX were assessed at 0, 0.5, 1, 2, 3, 4, 6, 8, 10, 12, 16, 24, 36, and 48 hours post-ticagrelor dose for pharmacokinetic analyses. Safety and tolerability was assessed throughout the study. Results: At 0.5 hours postdose, plasma concentrations of ticagrelor and AR-C124910XX were higher with crushed tablets administered orally (148.6 ng/mL and 13.0 ng/mL, respectively) or via nasogastric tube (264.6 ng/mL and 28.6 ng/mL, respectively) compared with whole-tablet administration (33.3 ng/mL and 5.2 ng/mL, respectively). A similar trend was observed at 1 hour postdose. Ticagrelor t_max_ was shorter following crushed vs. whole-tablet administration (1 vs. 2 hours, respectively). Geometric mean ratios between treatments for AUC and C_max_ were contained within the bioequivalence limits of 80 – 125% for ticagrelor and AR-C124910XX. All treatments were generally well tolerated. Conclusions: Ticagrelor administered as a crushed tablet is bioequivalent to whole-tablet administration, independent of mode of administration (oral or via nasogastric tube), and resulted in increased plasma concentrations of ticagrelor and AR-C124910XX at early timepoints.

## Introduction 

Ticagrelor is an orally-administered, direct-acting, reversibly-binding P2Y_12_ receptor antagonist used clinically for the prevention of atherothrombotic events in patients with acute coronary syndromes (ACS) [1, 2]. Ticagrelor mediates its antiplatelet effects via potent and reversible binding to the G-protein coupled P2Y_12_ receptor, which is expressed primarily on platelets [3]. In addition to its central mechanism of action, ticagrelor has also been shown to inhibit cellular uptake of adenosine via inhibition of the equilibrative nucleoside transporter 1 [4, 5]. Following oral administration, ticagrelor is rapidly absorbed and displays linear and predictable pharmacokinetics [6, 7]. Although ticagrelor is direct acting and exhibits a fast onset of action with respect to antiplatelet effect [2, 8], it is also metabolized by cytochrome P450 (CYP) 3A4 and 3A5 enzymes to its active metabolite AR-C124910XX [9], which is approximately equipotent with respect to inhibition of the P2Y_12_ receptor (AstraZeneca, data on file). 

The efficacy of ticagrelor was evaluated in 18,624 patients with ACS in the pivotal phase III Platelet Inhibition and Patient Outcomes (PLATO) study [10]. At 12 months, the primary composite endpoint of myocardial infarction, stroke, or death from vascular causes was reduced in patients receiving ticagrelor compared with those receiving clopidogrel (9.8% vs. 11.7%, respectively, hazard ratio (HR), 0.84; 95% confidence interval (CI), 0.77 – 0.92; p < 0.001), without an increase in overall major bleeding. Furthermore, treatment with ticagrelor was associated with a reduction in all-cause mortality compared with clopidogrel treatment. The current US Food and Drug Administration (FDA) recommendations for dosing and administration of ticagrelor include an oral loading dose of 180 mg followed by 90 mg twice daily (FDA Brilinta prescribing information [11]). 

Identifying novel methods of ticagrelor administration could benefit specific subsets of patients. For example, dysphagia is a common problem in elderly patients and in patients with cardiovascular disease, particularly in the hospital setting [12, 13]. These patients may have difficulty swallowing tablets and capsules and are more likely to experience medication administration errors [14]. Difficulties with swallowing are also reported in the nonacute setting, and are associated with efficacy and safety issues as well as nonadherence to treatment regimens [15]. Therefore, in patients who are unable to swallow a tablet whole and in patients with more severe dysphagia, it may be necessary to administer ticagrelor by crushing the tablet and dosing orally or by crushing and dosing directly into the stomach via a nasogastric tube. While the pharmacokinetics and pharmacodynamics of ticagrelor administered orally in whole-tablet form have been well documented [7, 16, 17], any of these alternative administration procedures might modify the pharmacokinetic properties of the drug. 

In order to assess the effect of alternative methods of administration on the bioavailability of ticagrelor, a clinical pharmacology study was conducted to investigate whether a crushed ticagrelor tablet administered orally or a crushed tablet given via nasogastric tube into the stomach were bioequivalent to a ticagrelor tablet swallowed whole. 

## Methods 

### Study population 

Healthy volunteers were to be between 18 and 50 years of age, have a body mass index (BMI) between 18 and 30 kg/m^2^, and weigh between 50 and 100 kg. Female volunteers of child-bearing potential were required to be using an effective form of birth control; those of nonchild bearing potential were required to be sterilized or postmenopausal. The main exclusion criteria included use of enzyme-inducing drugs within 3 weeks of the study start date, and any history of: hemophilia; von Willebrand’s disease; lupus anticoagulant or any other condition that may affect the propensity for bleeding; gastrointestinal, hepatic, or renal disease; or any other condition that may interfere with drug absorption, distribution, metabolism, or excretion. 

Based on findings from a previous study in which 18 volunteers received a single 90-mg ticagrelor dose, intrasubject variability for ticagrelor and AR-C124910XX pharmacokinetics (AUC and C_max_) were estimated to be ≤ 24% [18]. Assuming similar variability in the present study and a ratio of 1.0 for both AUC and C_max_, a sample size of 30 volunteers was required to show bioequivalence with 90% power at an α-level of 0.05. This would ensure the 90% CIs for the between-group ratios would be within the limits of 0.8 – 1.25. 36 volunteers were randomized to ensure data from at least 30 volunteers. 

All volunteers provided written, informed consent prior to study enrolment. The study protocol was reviewed and approved by an independent Ethics Committee (NHS Health Research Authority, NRES Committee East of England, Hatfield, UK). The study was performed in accordance with the Declaration of Helsinki and the International Conference on Harmonization/Good Clinical Practice Guidelines and, in addition, followed applicable regulatory requirements and AstraZeneca’s policy on bioethics. 

### Study design and treatments 

This study was a single-center, open-label, randomized, three-period, three-treatment crossover study (ClinicalTrials.gov identifier: NCT01887626; AstraZeneca study identifier: D5130C00076). Eligible volunteers were enrolled following a screening visit up to 28 days prior to day 1 of the study. The study comprised three treatment periods of 4 days during which volunteers received one of three treatments: ticagrelor 90-mg whole tablet administered as single oral dose with 200 mL of water, ticagrelor 90-mg tablet crushed and suspended in 200 mL water administered as single oral dose, or ticagrelor 90-mg tablet crushed, suspended in 200 mL water, and administered through a nasogastric tube into the stomach. As described previously [19], individual ticagrelor tablets were crushed using a mortar and pestle and transferred to a dosing cup. 100 mL of water was added to the mortar, stirred, and the contents were transferred to the dosing cup and stirred to form a suspension (ticagrelor has an aqueous solubility of 10 µg/mL [11]). The mortar was rinsed with a further 100 mL of water, and the contents were transferred to the dosing cup. Suspensions were prepared immediately prior to administration [19]. Following a wash-out period of at least 7 days, volunteers crossed over to receive the alternative treatments. 

Volunteers were required to fast from at least 10 hours prior to the planned ticagrelor dose on day 1 of the study. Volunteers were allowed up to 200 mL of water to drink up to 1 hour prior to drug administration, and then could resume drinking 2 hours after administration. The first meal was provided 4 hours after administration of the study drug. During the residential period at the study center, all subjects received standardized meals and drinks. Restricted activities during the study included: undergoing surgery, strenuous exercise, consumption of drinks containing alcohol, taurine, glucuronolactone, caffeine, or grapefruit, and use of tobacco- or nicotine-containing products, prescribed medication, aspirin, or nonsteroidal anti-inflammatory drugs. 

### Pharmacokinetic sampling and analytical methods 

To determine blood plasma concentrations of ticagrelor and its major metabolite, AR-C124910XX, blood samples were collected at 0 (predose), 0.5, 1, 2, 3, 4, 6, 8, 10, 12, 16, 24, 36, and 48 hours postdose during each treatment period. Plasma concentrations of ticagrelor and AR-C124910XX were quantified using fully validated reversed-phase liquid chromatography/tandem mass spectrometry [20]. For ticagrelor and AR-C124910XX respectively, lower limits of quantification were 5.0 and 2.5 ng/mL, mean intrabatch accuracy was 91.9 – 109.0% and 86.8 – 109.2%, and intrabatch precision was 4.0 – 8.4% and 5.2 – 16.9%. 

### Safety and tolerability assessments 

Safety and tolerability of ticagrelor administered as a crushed tablet either orally or via nasogastric tube were evaluated by assessment of the incidence of adverse events (AEs), various clinical parameters (clinical chemistry, hematology, and urinalysis), physical examinations, 12-lead electrocardiograms, and vital signs. 

### Data analyses 

Pharmacokinetic parameters were calculated by standard noncompartmental methods (WinNonlin Professional, Pharsight Corporation, Mountain View, CA, USA). Ticagrelor plasma concentration-time profile data were used to evaluate maximum plasma concentration (C_max_), time to C_max_ (t_max_), terminal elimination half-life (t_1/2_), area under the plasma concentration-time curve from time zero to infinity (AUC), and metabolite-to-parent ratios (for C_max_ and AUC) for ticagrelor and AR-C124910XX. t_1/2_ was calculated as 0.693/λ_z_; where λ_z_ is the terminal-phase elimination rate constant, estimated by least-squares regression analysis of the plasma concentration-time data. AUC was calculated as AUC_0–t_ plus C(t_n_)/λ_z_; where C(t_n_) was the final concentration observed, and AUC_0–t_ refers to the area under the plasma concentration-time curve from time 0 to the final measurable timepoint, calculated using the linear trapezoidal rule. 

Statistical analyses were performed using SAS version 9.2 (SAS Institute, Cary, NC, USA). Volunteers who received at least one study dose and had at least one postdose plasma concentration measurement were included in the analysis. Ticagrelor and AR-C124910XX pharmacokinetic parameters of AUC and C_max_ were natural log transformed and analyzed using a linear mixed-effects model with fixed effects for sequence, period, and treatment. Results were then back transformed and presented as geometric least-square means (95% CIs) and the ratios of geometric least-square means (GMR; 90% CIs). Ratios between treatments for AUC and C_max_ that were contained entirely within the limits of 80 to 125% were considered bioequivalent. In addition, the same model (with 95% CIs) was used to compare plasma concentrations of ticagrelor and AR-C124910XX at 0.5, 1, and 2 hours after administration of ticagrelor. 

## Results 

### Patient demographics and baseline characteristics 

36 volunteers were randomized to receive ticagrelor treatment. All volunteers completed the study and received the three treatments correctly; therefore, all 36 volunteers were included in the pharmacokinetic and safety analyses. None of the volunteers were prescribed concomitant medication during the study. 

Of the volunteers enrolled, 28 (77.8%) were male, 21 (58.3%) were White, 8 (22.2%) were Asian, 3 (8.3%) were Black or African American, 3 (8.3%) were Mixed and 1 (2.8%) was of African origin. The mean (± SD, range) age of volunteers was 31 (8, 20 – 49) years, and the mean weight and BMI were 74.5 (11.4, 55.2 – 94.0) kg and 24.4 (2.9, 19.4 – 29.6) kg/m^2^, respectively. 

### Pharmacokinetics 

**Figure 1:**
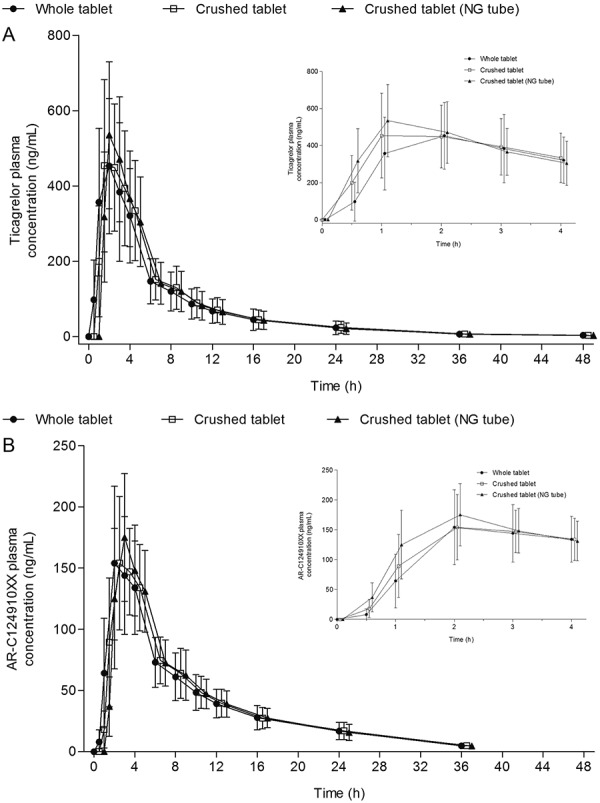
Figure 1. Arithmetic mean ± SD plasma concentrations of (A) ticagrelor and (B) AR-C124910XX over time following administration of a 90 mg dose of ticagrelor administered either orally as a whole tablet, orally as a crushed tablet, or as a crushed tablet via nasogastric tube.

Plasma concentration-time profiles of ticagrelor and its major metabolite AR-C124910XX are shown in Figure 1. Plasma concentrations of ticagrelor peaked at ~ 1 hour following ticagrelor 90 mg administered as a crushed tablet (median (range) t_max_ was 1 (1 – 4) hour and 1 (1 – 3) hour when administered orally or via nasogastric tube, respectively). When 90 mg ticagrelor was administered orally as a whole tablet, peak plasma concentrations were observed at 2 hours (median t_max_ 2 (1 – 4) hours). Plasma concentrations of AR-C124910XX peaked at ~ 2 hours with all treatments (median t_max_ 2 (1 – 8) hours) (Table 1). 

At 0.5 hours postdose, plasma concentrations of ticagrelor were higher with crushed ticagrelor tablets administered orally (148.6 ng/mL) or via nasogastric tube (264.6 ng/mL) compared with whole-tablet administration (33.3 ng/mL) (Table 2). The GMR of ticagrelor plasma concentrations was significantly higher with crushed ticagrelor tablets, administered either orally (445.9%, 95% CI, 243.0 – 818.0) or via nasogastric tube (793.7%, 95% CI, 432.7 – 1,456.1), compared with whole-tablet administration. Similarly, plasma concentrations of AR-C124910XX were significantly higher following administration of crushed tablets either orally (13.0 ng/mL; GMR, 247.6%, 95% CI, 169.9 – 360.7) or via nasogastric tube (28.6 ng/mL; GMR, 546.6%, 95% CI, 375.1 – 796.5) compared with whole-tablet administration (5.2 ng/mL) (Table 2). At 1 hour postdose, a similar trend to that seen at 0.5 hours was observed for both ticagrelor and AR-C124910XX plasma concentrations (Table 2). Between 2 and 48 hours post-dose, and 3 and 48 hours postdose, plasma concentration-time profiles for both ticagrelor and AR-C124910XX (respectively) were comparable between treatments (Figure 1). 

No significant differences in AUC and C_max_ were observed between treatments. The GMRs (90% CIs) of AUC and C_max_ between treatments were contained within the prespecified limits of 80 – 125% for bioequivalence for both ticagrelor and AR-C124910XX (Table 1), indicating that overall exposure to ticagrelor or AR-C124910XX were unaffected by the method of administration. The AUC for ticagrelor and AR-C124910XX were well estimated, with the extrapolated portion representing < 5% of the total AUC for ticagrelor and < 15% of the total AUC for AR-C124910XX for all volunteers across all treatments. 

### Safety and tolerability 

All ticagrelor treatments were generally well tolerated. There were no deaths or serious AEs in the study, and no volunteers were discontinued due to an AE. Overall, 16/36 (44.4%) volunteers experienced at least one AE. The most common AEs were nervous system disorders (dizziness, headache, migraine, presyncope, and syncope) and infections (oral herpes, tooth infection, and upper respiratory tract infections), which occurred in 13.9% and 11.1% of volunteers, respectively. All AEs were mild in intensity with the exception of 2 cases. One volunteer reported tooth infection following oral administration of crushed ticagrelor, which was considered moderate in intensity. Another volunteer reported moderate migraine, also following oral administration of crushed ticagrelor. Both moderate AEs resolved before the end of the study and were considered unrelated to the treatment. No clinically relevant changes in clinical laboratory parameters, physical examinations, vital signs, or electrocardiographies (ECGs) were observed. 

## Discussion 

The pharmacokinetic and pharmacodynamic profiles of ticagrelor administered as a whole tablet are well documented [7, 16, 17]. However, the effects of administering ticagrelor via alternative methods have not previously been assessed. Therefore, this study examined whether a crushed ticagrelor tablet administered orally or as a crushed tablet given via nasogastric tube were bioequivalent to a ticagrelor tablet swallowed whole. 

In the present study, the pharmacokinetic profile observed for a single 90-mg dose of ticagrelor administered orally as a whole tablet, including a t_max_ in the region of 2 hours and t_1/2_ of ~ 8 hours, was broadly comparable with that reported in previous studies in healthy volunteers [7]. Compared with administration of ticagrelor as a whole tablet, administration as a crushed tablet (either orally or via nasogastric tube) resulted in significantly higher plasma concentrations of ticagrelor and AR-C124910XX at early timepoints (0.5 and 1 hour). Furthermore, maximum plasma concentrations of the parent compound were achieved earlier following administration of ticagrelor as a crushed tablet (1 hour) vs. whole tablet (2 hours). The method of administration had no significant effect on overall exposure to ticagrelor or AR-C124910XX as assessed by C_max_ and AUC. The GMRs of C_max_ and AUC between treatments were contained within the prespecified limits of bioequivalence, indicating that ticagrelor can be given as a whole tablet (orally) or a crushed tablet (either orally or via nasogastric tube) without dose modification. 

Based on these results, crushed tablets may represent a viable alternative method of administering ticagrelor. While further studies are warranted to examine the pharmacodynamic effects of administering ticagrelor as a crushed tablet (e.g., effects on platelet aggregation, bleeding times, etc.), the present findings suggest that the alternative administration methods studied here could potentially offer clinical benefits. For example, dysphagia is a common problem in the hospital setting [12, 13], and the option to administer ticagrelor in crushed-tablet form would likely benefit patients with this condition who have difficulty swallowing whole tablets or capsules. Furthermore, in the acute setting, where a patient may not always be conscious, administration of ticagrelor as a crushed tablet directly into the stomach via nasogastric tube could offer an effective method of drug delivery. 

Crushed tablet preparations could also be evaluated as a method of providing a faster onset of platelet inhibition. This application may offer clinical benefit in certain patient subsets; for example in ST-segment-elevation myocardial infarction (STEMI) patients, a delay in the onset of ticagrelor’s (and prasugrel’s) antiplatelet effects, possibly due to concomitant morphine administration or as a physiologic consequence of acute myocardial infarction has been documented [21, 22, 23]. The MOJITO study is currently evaluating platelet reactivity following administration of crushed vs. intact ticagrelor loading doses in STEMI patients (clinicaltrials.gov NCT01992523). 

With regards to safety and tolerability, all preparations of ticagrelor were generally well tolerated regardless of the method of administration. No serious AEs were reported, and no patients were discontinued from the study due to an AE. Most AEs that occurred were mild in intensity and consistent with the previously reported safety profile of ticagrelor [7]. 

In conclusion, administration of ticagrelor as a crushed tablet orally or via nasogastric tube was associated with increased plasma concentrations of ticagrelor and AR-C124910XX at early timepoints compared with whole-tablet administration. Regardless of the method of administration, crushed preparations of ticagrelor were bioequivalent to whole-tablet preparations. 

## Acknowledgments 

Medical writing support was provided by Lisa Michel and David Evans (Gardiner-Caldwell Communications, part of the Kowledgepoint360 group, an Ashfield company) and was funded by AstraZeneca. 

## Conflict of interest 

The authors are employees of AstraZeneca and may own stock. 

**Table 1:** Pharmacokinetic parameters and exposure ratios of ticagrelor and AR-C124910XX following administration of a 90-mg dose of ticagrelor either orally as a whole tablet, orally as a crushed tablet, or as a crushed tablet via nasogastric tube.

	Ticagrelor (90 mg)			Ratio of geometric means (90% CI)^b^	
Parameter^a^	Whole tablet n = 36	Crushed tablet n = 36	Crushed tablet (NG tube) n = 36	Crushed vs. whole tablet	Crushed (NG tube) vs. whole tablet
Ticagrelor					
C_max_ (ng/mL)	506 (35.8)	529 (37.0)	547 (37.5)	104.4 (97.5 – 111.9)	108.00 (100.8 – 115.7)
AUC (ng×h/mL)	2,890 (43.8)	3,090 (43.7)	3,050 (40.5)	107.2 (102.5 – 112.0)	105.8 (101.2 – 110.6)
AUC_0–t_ (ng×h/mL)	2,840 (43.8)	3,050 (43.4)	3,020 (40.1)	–	–
t_1/2_ (hours)	7.56 (1.2)	7.79 (1.2)	7.40 (1.2)	–	–
t_max_ (hours)	2.0 (1.0 ‒ 4.0)	1.0 (1.0 ‒ 4.0)	1.0 (1.0 ‒ 3.0)	–	–
AR-C124910XX					
C_max_ (ng/mL)	165 (30.1)	166 (25.3)	174 (28.2)	100.8 (93.9 – 108.2)	105.9 (98.7 – 113.7)
AUC (ng×h/mL)	1,380 (30.7)	1,430 (27.7)	1,460 (25.4)	103.3 (99.5 – 107.3)	105.2 (101.3 – 109.3)
AUC_0–t_ (ng×h/mL)	1,320 (32.6)	1,370 (29.2)	1,410 (26.6)	–	–
t_1/2_ (hours)	8.30 (1.2)	8.32 (1.2)	7.95 (1.2)	–	–
t_max_ (hours)	2.0 (2.0 ‒ 4.0)	2.0 (2.0 ‒ 8.0)	2.0(1.0 ‒ 4.0)	–	–
Metabolite : parent ratios					
C_max_ ratio	0.325 (31.3)	0.314 (24.0)	0.319 (27.7)	–	–
AUC ratio	0.480 (34.0)	0.463 (28.3)	0.477 (30.3)	–	–

^a^Values are: geometric mean (% CV) for C_max_, AUC, and metabolite : parent ratios; mean (standard deviation) for t_1/2_; median (range) for t_max_; ^b^Ratios of geometric means are presented as percentages. CI = confidence intervals; C_max_ = maximum plasma concentration; AUC = area under the plasma concentration-time curve from time 0 to infinity; t_1/2_ = terminal elimination half-life; t_max_ = time to C_max_; NG = nasogastric.

**Table 2:** Plasma concentrations of ticagrelor and AR-C124910XX at 0.5, 1, and 2 hours postadministration of a 90-mg dose of ticagrelor either orally as a whole tablet, orally as a crushed tablet, or as a crushed tablet via nasogastric tube.

Timepoint	Geometric mean plasma concentrations (95% CI)		
	Whole tablet n = 36	Crushed tablet n = 36	Crushed tablet (NG tube) n = 36
Ticagrelor (ng/mL)			
0.5 hour	33.3 (21.7 – 51.2)	148.6 (96.8 – 228.3)	264.6 (172.3 – 406.3)
1.0 hour	270.9 (216.6 – 338.8)	392.5 (313.9 – 490.9)	497.8 (398.1 – 622.5)
2.0 hours	414.7 (360.1 – 477.6)	418.4 (363.3 – 481.8)	442.1 (383.9 – 509.1)
AR-C124910XX (ng/mL)			
0.5 hour	5.2 (4.0 – 6.9)	13.0 (9.9 – 17.0)	28.6 (21.8 – 37.6)
1.0 hours	41.4 (31.3 – 54.6)	73.2 (55.5 – 96.7)	111.8 (84.8 – 147.6)
2.0 hours	141.8 (125.8 – 159.7)	143.8 (127.7 – 162.1)	167.9 (149.0 – 189.2)

CI = confidence interval; NG = nasogastric.
